# Educational attainment and mortality: results from the Sixth Population Census in China

**DOI:** 10.7189/jogh.09.020604

**Published:** 2019-12

**Authors:** Shangcheng Zhou, Guanyang Zou, Xiaping Chen, Hongxing Yu, Jing Wang, Pengqian Fang, Fujian Song

**Affiliations:** 1 School of Economics and Management, Guangzhou University of Chinese Medicine, Guangzhou, Guangdong, China; 2 Shiyan Taihe hospital, Hubei University of Medicine, Shiyan, Hubei, China; 3 Center of Health Administration and Development Studies, Hubei University of Medicine, Shiyan, Hubei, China; 4 Tongji Medical College, Huazhong University of Science and Technology, Wuhan, Hubei, China; 5 Faculty of Medicine and Health Science, University of East Anglia, Norwich, UK

## Abstract

**Background:**

Health and education are two closely related factors that affect human development. A limited number of studies have been conducted in China, most of which have been based on small sample sizes and with inconsistent results. The study investigates the association between mortality rate and educational level in China based on the sixth national population census in 2010.

**Methods:**

This is large-scale population study based on the nationally administrated data sets of population census in 2010, 2000 and 1990. The 2010 census covered a total population of 1 332 810,869 in China.

**Results:**

In general, standardized mortality rate decreased as educational level increased. The standardized mortality rate is higher among males than among females with equivalent educational levels. The standardized mortality rate in all the educational groups declined to varying degrees from 1990. 2000 to 2010. The standardised mortality rate declined with increasing educational levels from no education to university undergraduate groups in 1990, 2000 and 2010. The standardized mortality rate declined as the degree of education increased in cities, towns, and villages, but gradually increased at the same educational level from cities, towns, to villages in general. The difference in each region is considerable and the population quality of the developed area is generally high. The percentage of the uneducated population to the total population aged 15 years and over (%) was positively correlated with the standardized mortality rate. By contrast, the percentage of the population with a high school education to the total population aged 6 years and over (%) was negatively correlated with the standardized mortality rate.

**Conclusions:**

We found that educational level was negatively correlated with the mortality rate. The crude and standardized death rate is lower among individuals with higher educational level. Together with previous research findings, this study indicates that improving total population education attainment remains an important challenge that requires imperative action, while reducing educational inequities remains crucial for the government.

Non-medical factors, such as education, family, and environment, play an important role in health than health care investment [1]. Health and education are two closely related factors that affect human development. Previous studies have found that people with higher educational levels are healthier and enjoy increased longevity [2-4]. Grossman and Kaestner [5] found that school education was negatively correlated with morbidity and mortality, and positively correlated with self-assessment of health. A recent study reported a strong correlation between the mortality rate and educational level in the United States [6]. However, Galam et al. reported in 2018 that education was only effective on mortality in some contexts, and a number of other socio-economic factors also contributed to this effect, such as gender, quality of education and the labor market returns to education, influence of education on quality of individuals’ peers [7]. To date, most studies on the relationship between mortality rate and educational level have been conducted in Western countries [8,9], with inconsistent conclusions. For example, one study showed that the turnaround in mortality for white non-Hispanics was driven primarily by increasing death rates for those with a high school degrees or less [10]. A limited number of studies have been conducted in China, most of which have been based on small sample sizes and with inconsistent results [11-14]. For instance, one study in China found that people with higher educational levels tended to die prematurely [15]. Another study found an inverse relationship between education and mortality risk among older adults in China, using data from the 2002 to 2011 waves of the Chinese Longitudinal Healthy Longevity Survey [16].

Chinese education is mainly run by the State. All citizens must attend the compulsory education that includes six years of primary education, starting at age six or seven, and three years of junior middle school for ages 12 to 15. This is followed by three years of senior middle school education, and the students can continue to study in the college and universities if they can pass the colleague entrance examinations, and the education can be up to the postgraduate level (masters and PhD).

While the evidence on relationship between educational level and mortality rate remains scarce, there have been very few studies that use census data to examine such relationship in China [17]. Census data are used as an authoritative source of information to estimate the death rates in populations. Population censuses rely on strict individual registration systems and are deemed to be highly credible. China started to conduct the population census since 1953, roughly every ten years. The third population census (known as 1982 census), was the first ever census to collect the mortality data. By 2010, China has conducted six population census. Census data encompass educational level, gender, age, and mortality rate for the population of China, thereby allowing researchers to investigate differences in the mortality rates of various population subgroups in China.

In this study, we used data from the 2010 census (the sixth population census) to analyze the relationship between educational level and mortality rate, taking into consideration gender, age, region, time, and other influential factors.

## METHODS

### Data

We collected three nationally administrated data sets of population census. The first was the China’s sixth national population census, administered between November 1, 2009 and October 30, 2010, from the Chinese government’s census website: (http://www.stats.gov.cn/tjsj/pcsj/rkpc/6rp/indexch.htm). We also collected data on the 1990 (fourth) and 2000 (fifth) national population census published by the Chinese Statistics Press. We mainly used the data from 2010 census year for analysis, and where appropriate, we compared this data with that in 1990 and 2000. The 2010 census committee has reported that the quality of census data (for the) is very high, with a false negative rate of 0.12%. The population Census was based on household interviews. The Census officer filled in the census form item by item on the site, while ensuring the accuracy and completeness of the data. The residents signed to confirm the accuracy of the data after the interview. The Census supervisor rechecked the census results and went to the households again to correct the data. The State Council conducted the sampling check of the data afterwards (http://www.stats.gov.cn/tjsj/pcsj/rkpc/6rp/html/fu07.htm).

### Measures

#### Mortality

Demographers have found that two factors determine the crude death rate. The first factor is the age-specific mortality rate, an index of the number of deaths. The second factor is the age structure of the population, which is independent of the mortality rate. A standardized mortality rate is important to measure the differences in the number of deaths between regions. The crude death rate is typically affected by population distribution, population age structure, and gender structure. To eliminate the influences of age composition, we standardized all the educated populations (including gender populations), based on the total standard population in 2010. We used standardization methods to control for the influences of age and gender, and possibly, other factors, on the comparison of mortality rates between two or more populations.

#### Indirect age-adjustment standardization

In indirect age-adjustment standardization, a common set of age-specific rates is applied to populations whose rates have to be standardized. This indirect adjustment method is used for regional population analysis. The simplest and most useful form of indirect adjustment is the standardized mortality ratio (SMR) [18], which is calculated as SMR = Number of Observed Deaths/Number of Expected Deaths(Expected Deaths = Σ(Standard age-specific death rate) × (Study age-specific pop weight)). Standardized mortality rate = standard population mortality × SMR formula. In this study, we adopted the national age-specific population in 2010 as the standard.

Direct age-adjustment standardization. In this study, we adopted the national average age in 2010 as the standard and used the direct method to calculate the standardized mortality rates in various conditions. We used this direct adjustment method for all analyses except regional population analysis. The specific steps were as follows:

(a) We multiplied the standard population composition of each age group by the age group of the original mortality rate. The results were the two parts of the age group based on the standard population calculation of the expected number of deaths.

(b) We calculated the summation in each age group using the expected number of deaths in the standard population. We divided the expected total number of deaths by the total population standard. We called the result the standard mortality.

#### Data processing of special death population data

The 1990 census measured the population in the middle of 1990, whereas it calculated the number of deaths using data for the first three months of the census. Thus, the population was not concordant with the number of deaths in 1990. The 1990 mortality rate denotes the number of deaths for two-thirds of the 18 months (ie, January 1, 1989 to June 30, 1990) as the numerator, with number of deaths for one-third of the 18 months plus the total population in the 1990 census as the mean population estimates (ie, the denominator) before the census year.

#### Educational attainment

The generally accepted measure of formal education comprises statistics from the age of 6 years. The 2010 census classified educational level into seven groups: no school, primary school, junior high school, high school, college, university undergraduate, and postgraduate. However, the census year classification varied slightly in its analysis of the difference in urban–rural and regional mortality rates. Every year, it merged high school and secondary school populations and classified the uneducated population under no school.

### Statistical analyses

The data analysis involves a few steps. First, we conducted descriptive analysis about the general characteristics of the population census. Second, we analysed the relationship between the gender and educational attainment using the 2010 census data, mainly using single factor non-conditional logistic regression analysis. Third, we analysed the mortality difference between the three population census: 1990, 2000 and 2010. It is worth mentioning that the 1990 census did not include the graduate level in the educational levels. The 2000 census added the graduate level classification and did not use separate categories for the no education and uneducated classes. To facilitate the comparison of classification standards, we regarded the uneducated class in the 1990 census as equivalent to the no education class. We divided annual educational levels into seven categories. Fourth, we anlaysed the mortality difference between city, urban and rural areas. The city area refers to communities and other areas that are actually connected to the district and city governments in municipal districts, and smaller cities without districts. Town area refers to the communities and other areas outside city areas that are actually connected to government garrison areas in county government and other towns. The following areas that are not actually connected to the government located areas with permanent populations over 3000 people are also regarded as towns: resident areas of independent industrial and mining sites, development zones, scientific research units, universities, as well as the yard of farms and forest farms. The country side population refers to all the other people except the city and town population. Fifth, we anlaysed the correlation between educational attainment and mortality and liner regression analysis was conducted to confirm the effect of two factors on the standardized mortality rate: 1) the percentage of the uneducated population to the total population aged 15 years and over and 2) the percentage of the population with a high school education to the total population aged 6 years and over .Finally, using cluster analysis, we measured the population quality, which is typically defined based on three elements: physical quality, educational quality, and moral quality. However, given the available data, our definition of population quality included only two factors: mortality rate as the representative of physical quality and educational attainment as the indicator of educational quality percentage of the uneducated population to the total population aged 15 years and over, percentage of the population with a high school education to the total population aged 6 years and over. SPSS software (version 16.0) was used for analysis.

## RESULTS

### General characteristics of the population census

**Figure 1:**
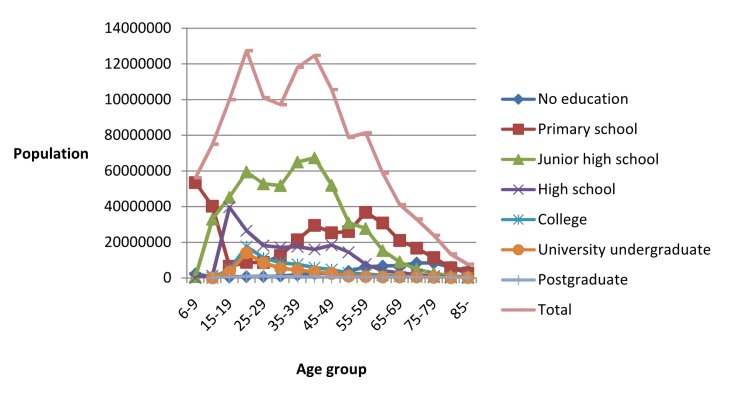
Educational attainment of the population in 2010 by age group for 6 years old and above.

The population census in 2010 covered a total population of 1 332 810,869, including 682 329 104 males (51%) and 650 481 765 females (49%). **Figure 1** shows the population concentration in 2010. The 20-44 age group was the largest group, accounting for 42% of the total population. The educational attainment was mainly junior high school/primary school, and only a few had a college degree or higher.

### Gender and educational attainment: pooled sixth census population data in 2010

**Table 1** shows that older individuals had lower educational levels, and the crude mortality rate of the uneducated population was considerably higher than that of the educated population. As educational levels improved, the crude mortality rate significantly declined. The mortality rate of the uneducated population was five times higher than that of the educated population. The single factor non-conditional logistic regression analysis showed that lower education level (Wald *x*^2^ = 11.848, *P* = 0.001) and male (Wald *x*^2^ = 11.359, *P* = 0.001) were possible risk factors for higher mortality.

**Table 1:** Crude mortality rate and standardized mortality rate of the population aged 6 y and above (‰)

Educational attainment	No standardization			With standardization			
**Total**	**Male**	**Female**	**Total**	No education/X*	**Male**	**Female**
No education	42.13	56.70	36.42	7.99	1	12.00	6.63
Primary school	8.62	11.87	5.73	5.89	1.36	7.65	4.14
Junior high school	2.45	3.49	1.24	5.05	1.58	6.31	3.06
High school	1.42	1.99	0.70	3.09	2.59	3.85	1.81
College	0.77	1.12	0.36	1.89	4.23	2.26	1.18
University undergraduate	0.66	0.95	0.30	1.62	4.93	1.90	1.03
Postgraduate	0.28	0.36	0.17	1.11	7.20	1.22	0.89
Total	5.93	6.72	5.10	5.93	——	7.27	4.73

*X indicates each educational attainment. For example, 7.99/5.89 **=** 1.36, 7.99/5.05 **=** 1.58, and so on.

In general, standardised mortality rate clearly decreased as educational level increased. The mortality rate declined more rapidly for populations with high school or higher education levels, which reflects the considerable influence of high school and higher education on population mortality rates. The standardised mortality rate of the uneducated population was 7.20, 4.93, 4.23, 2.59, 1.58, and 1.36 times higher than populations with a postgraduate degree, with an undergraduate degree, with a high school education, with a middle school education, and with a primary school education, respectively.

With the increase in educational level, the gap in the crude mortality rate between males and females initially expanded and then narrowed, following a U-shape trend. The crude death rate of males decreased rapidly compared to that of females.

### Mortality difference between 1990, 2000 and 2010

**Table 2** shows that, across time from 1990 to 2010, the crude mortality rate of the populations with low levels of education (between no education and junior high school) increased for both gender groups. The mortality for other educational groups except the group with senior high school education decreased across years from 1990 to 2010 for both gender groups. The crude mortality increased with decreasing educational levels for men (from high school to no education) and for women (from college to no education) in 1990, 2000 and 2010.

**Table 2:** Actual mortality rate of the population aged 6 y and above (‰)

Educational attainment	Total			Male			Female		
**2010**	**2000**	**1990**	**2010**	**2000**	**1990**	**2010**	**2000**	**1990**
No education	42.13	30.00	18.75	56.70	40.74	25.11	36.42	25.68	15.79
Primary school	8.62	5.49	3.72	11.87	7.90	5.49	5.73	3.17	1.76
Junior high school	2.45	2.04	1.81	3.49	2.81	2.35	1.24	1.04	0.99
High school	1.42	1.60	1.37	1.99	2.20	1.77	0.70	0.78	0.74
College	0.77	1.21	1.49	1.12	1.70	1.87	0.36	0.49	0.66
University undergraduate	0.66	1.45	2.07	0.95	1.85	2.45	0.30	0.70	1.11
Postgraduate	0.28	0.82	–	0.36	0.93	–	0.17	0.55	–
Total	5.93	5.94	6.09	6.72	6.57	6.55	5.10	5.29	5.59

**Table 2** and **Table 3** show the differences in the crude and standardized rates. The standardised rate for people with no education in all the given years and the standardized rate for people with primary school education in 2010 were lower than the crude rate in corresponding educational groups. The standardized rates of other educational groups were higher than the crude rates in the corresponding educational groups.

**Table 3:** Standardized mortality rate of the population aged 6 y and above (‰)

Educational attainment	Total			Difference		Male			Female		
**2010**	**2000**	**1990**	**2010/1990**	**2000/1990**	**2010**	**2000**	**1990**	**2010**	**2000**	**1990**
No education	7.99	8.03	8.68	0.69	0.65	12.00	10.80	11.18	6.63	6.90	7.45
Primary school	5.89	7.00	8.62	2.73	1.62	7.65	8.26	9.36	4.14	5.12	6.77
Junior high school	5.05	6.26	7.86	2.81	1.6	6.31	7.15	8.44	3.06	4.37	6.30
High school	3.09	4.25	5.62	2.53	1.37	3.85	4.98	6.32	1.81	2.72	3.97
College	1.89	2.92	4.17	2.28	1.25	2.26	3.33	4.58	1.18	1.78	2.71
University undergraduate	1.62	2.41	3.36	1.74	0.95	1.90	2.68	3.72	1.03	1.61	2.19
Postgraduate	1.11	2.44	–	–	–	1.22	2.52	–	0.89	2.14	–
Total	5.93	7.09	8.46	2.53	1.37	7.27	8.27	9.81	4.73	5.98	7.23

**Table 3** further shows that with the change in time, the standardized mortality rate in all the educational groups declined to varying degrees from 1990 to 2010. The decline in the male mortality rate was the fastest in the population with a high school education, whereas the decline in the female mortality rate was the fastest in the population with a junior high school education. The male mortality rate for the population with no education increased with time, whereas that of female declined. The standardised mortality rate decreased with increasing educational levels from no education to university undergraduate groups in 1990, 2000 and 2010.

The gap of the standardized mortality rate between 2000 and 1990 was comparatively smaller than the gap between 1990 and 2010. The differences in standardized mortality rate of the population with primary and secondary education declined further between 2000 and 1990 and between 1990 and 2010.

### Mortality difference between city, urban and rural areas

**Table 4** shows that crude mortality rate reduced with the increase of educational level whether in city, town, or rural areas. The crude total mortality rate declined from that of rural population, town population to city population. From primary school to postgraduate groups, the crude total and gender-related mortality rates of towns were lower than those of the cities and rural areas.

**Table 4:** Actual mortality rate of city/town/countryside population (‰)

Educational attainment	City			Town			Countryside		
**Total**	**Male**	**Female**	**Total**	**Male**	**Female**	**Total**	**Male**	**Female**
No school	38.83	49.28	35.39	39.86	54.45	34.45	43.25	58.32	37.08
Primary school	8.60	10.83	6.65	7.69	10.58	5.15	8.89	12.49	5.65
Junior high school	2.55	3.54	1.49	2.14	3.12	1.05	2.52	3.59	1.19
High school	1.43	2.00	0.81	1.11	1.60	0.49	1.68	2.30	0.66
College	0.76	1.12	0.38	0.72	1.05	0.29	0.89	1.28	0.37
University undergraduate	0.72	1.04	0.34	0.42	0.62	0.16	0.55	0.76	0.27
Postgraduate	0.21	0.29	0.11	0.37	0.41	0.32	2.27	2.61	1.86
Total	3.61	4.12	3.08	4.74	5.42	4.02	7.79	8.83	6.72

**Table 5** suggests that, after standardization, the overall mortality rate declined in the countryside, whereas those of the towns and cities increased, as compared to crude rates.

**Table 5:** Standardized mortality rate of city/town/countryside population (‰)

Educational attainment	City			Town			Countryside			Mortality difference* (total)	
**Total**	**Male**	**Female**	**Total**	**Male**	**Female**	**Total**	**Male**	**Female**	**Town**	**Countryside**
No education	6.15	9.87	5.18	7.26	10.83	6.05	8.58	12.03	7.22	1.97	2.18
Primary school	4.89	6.15	3.84	5.29	6.56	4.00	6.40	7.65	4.90	0.89	0.14
Junior high school	3.94	4.57	2.96	4.40	4.96	3.21	6.26	6.71	4.79	1.63	1.72
High school	2.70	3.13	2.00	2.77	3.03	1.94	4.54	4.52	3.57	0.86	1.21
College	1.70	1.85	1.31	1.91	2.00	1.44	3.33	3.22	3.17	0.36	0.37
University undergraduate	1.36	1.70	1.20	1.55	1.62	1.12	2.96	2.90	2.82	0.07	−2.54
Postgraduate	0.86	0.86	0.76	1.48	1.25	1.96	5.50	6.15	4.62	-	-
Total	4.15	4.65	3.62	5.24	5.91	4.52	7.04	8.05	5.99	-	-

*Mortality difference = lower education mortality – next higher education mortality.

The standardized mortality rate declined as the degree of education increased in cities, towns, and villages, but gradually increased at the same educational level from cities to towns and villages in general. However, there are exceptions. For example, the male and female mortality of high school and undergraduate in the town was lower than that of the same educational groups in the city. The mortality rate of postgraduates increased after a declining trend with the increased educational level in the rural area, and this was much higher than that of postgraduates in city and town- this may be an unstable result due to the relatively small sample size in the postgraduate group.

**Table 5** presents the mortality rate difference (mortality of lower education group – mortality of next higher education group). Primary school (1.26 per thousand) and high school (1.24 per thousand) have the most significant increase of the mortality rate in the city. This result also indicates that the mortality rate of the university degree group decreased slowly (0.34 per 1000) in the city. Moreover, the mortality rate of the postgraduate degree group decreased most slowly (0.07 per thousand) in the town. The slowest decline occurred in the village population with a junior high school education (0.14 per thousand).

According to “Provisional Regulations on the statistical division of urban and rural areas (2008)” by National Bureau of Statistics of China, urban area includes cities and towns. Table 6 showed the mortality rate difference between urban and rural by gender. In general, the crude and standardized urban mortality rate is lower than rural mortality rate at the same education level, except the crude mortality rate in the university undergraduate group.

**Table 6:** Urban-rural population standardization and actual mortality (‰)

Educational attainment	Standardization mortality(‰)						Actual mortality(‰)					
**Urban**			**Rural**			**Urban**			**Rural**			
**Total**	**Male**	**Female**	**Total**	**Male**	**Female**	**Total**	**Male**	**Female**	**Total**	**Male**	**Female**	
No education	6.69	10.42	5.62	8.58	12.03	7.22	39.40	52.25	34.88	43.25	58.32	37.08	
Primary school	5.05	6.35	3.91	6.40	7.65	4.90	8.14	10.70	5.88	8.89	12.49	5.65	
Junior high school	4.08	4.72	3.04	6.26	6.71	4.79	2.37	3.36	1.30	2.52	3.59	1.19	
High school	2.70	3.10	1.98	4.54	4.52	3.57	1.33	1.86	0.71	1.68	2.30	0.66	
College	1.73	1.89	1.33	3.33	3.22	3.17	0.75	1.10	0.36	0.89	1.28	0.37	
University undergraduate	1.57	1.69	1.19	2.96	2.90	2.82	0.67	0.96	0.31	0.55	0.76	0.27	
Postgraduate	0.89	0.89	0.84	5.50	6.15	4.62	0.22	0.29	0.12	2.27	2.61	1.86	
total	4.61	5.18	4.00	7.04	8.05	5.99	4.05	4.63	3.45	7.79	8.83	6.72	

### Educational attainment and mortality rate of a population: correlation and regression

**Table 7** shows the relationship between the regional educational level indicators and the standardized mortality rate. Both the percentages of the uneducated population to the total population aged 15 years and over (%) and the population with a high school education to the total population aged 6 years and over (%) significantly affected the standardized mortality rate (**Table 8**). The percentage of the uneducated population to the total population aged 15 years and over (%) was positively correlated with the standardised mortality rate. By contrast, the percentage of the population with a high school education to the total population aged 6 years and over (%) was negatively correlated with the mortality rate. We found the strongest correlation in males with a high school education aged 6 years and over (%) (coefficient −0.810).

**Table 7:** Regional educational level indicators and standardized mortality rate of the population

Regions	Percentage of uneducated population to the total population aged 15 y and above (%)			Percentage of population with a high school education to the total population aged 6 y and above (%)			Standardized mortality rate (‰)		
	**Male**	**Female**	**Total**	**Male**	**Female**	**Total**	**Male**	**Female**	**Total**
Total	2.52	7.29	4.88	26.6	22.42	24.55	6.29	4.81	5.57
Beijing	0.79	3	1.86	54.43	55.52	54.96	4.83	3.20	3.99
Tianjin	0.99	3.86	2.33	39.51	40.2	39.83	4.75	3.58	4.16
Hebei	1.58	4.71	3.14	22.89	20.55	21.73	8.08	5.35	6.66
Shanxi	1.48	3.72	2.57	27.12	24.93	26.05	7.53	5.11	6.30
Inner Mongolia	2.69	6.93	4.73	27.96	25.56	26.81	6.58	4.34	5.48
Liaoning	1.1	3.27	2.18	28.97	26.9	27.95	6.65	4.47	5.54
Jilin	1.35	3.03	2.18	29.17	27.02	28.1	5.94	4.16	5.04
Heilongjiang	1.39	3.31	2.34	26.25	24.24	25.26	6.40	4.14	5.26
Shanghai	1.12	4.99	3	46.36	42.86	44.66	4.61	2.96	3.73
Jiangsu	1.85	6.85	4.36	32.18	25.05	28.62	6.27	4.06	5.10
Zhejiang	3.28	9.8	6.47	25.96	22.37	24.21	5.94	3.87	4.90
Anhui	5.38	14.39	9.9	21.91	15.99	18.98	6.66	4.24	5.42
Fujian	1.07	4.76	2.89	26.74	20.86	23.87	6.57	4.07	5.30
Jiangxi	1.72	6.35	4.02	24.69	17.43	21.14	7.50	4.61	6.01
Shandong	2.86	8.92	5.89	27.21	21.22	24.23	6.92	4.39	5.58
Henan	2.96	7.7	5.37	23.49	19.61	21.55	7.26	4.43	5.74
Hubei	2.57	8.14	5.32	30.79	24.77	27.85	6.48	4.35	5.39
Hunan	1.64	4.89	3.24	27.19	22.53	24.91	6.48	4.31	5.38
Guangdong	0.88	4.03	2.41	30.64	24.78	27.83	6.43	3.88	5.12
Guangxi	1.4	5.64	3.46	20.4	16.91	18.71	7.05	3.77	5.36
Hainan	2.06	8.35	5.07	28.11	20.76	24.63	5.37	2.98	4.16
Chongqing	2.76	7.4	5.08	24.55	21.86	23.22	6.53	4.20	5.36
Sichuan	3.78	9.37	6.55	20.58	17.67	19.15	7.03	4.61	5.81
Guizhou	5.71	17.26	11.4	15.69	12.31	14.04	8.14	5.21	6.66
Yunnan	4.41	11	7.6	16.06	14.24	15.18	8.94	5.89	7.39
Tibet	24.22	40.86	32.29	12.09	9.75	10.95	9.22	6.94	7.98
Shaanxi	2.48	6.4	4.39	29.93	25.8	27.93	6.85	4.95	5.90
Gansu	6.5	14.83	10.62	24.54	18.57	21.61	7.37	5.37	6.37
Qinghai	8.04	18.2	12.94	22.13	19.1	20.67	8.33	6.20	7.27
Ningxia	4.51	11.24	7.82	25.46	22.06	23.8	8.32	6.01	7.18
Xinjiang	2.22	3.85	3.01	24.6	24.08	24.35	6.32	4.62	5.51

**Table8:** Correlation between educational attainment and mortality rate

Standardized mortality rate	Percentage of uneducated population to the total population aged 15 y and above (%)			Percentage of the population with a high school education to the total population aged 6 y and above (%)		
**Male**	**Female**	**Total**	**Male**	**Female**	**Total**
Male	0.588*			−0.810*		
Female		0.669*			−0.599*	
Total			0.644*			−0.740*

*P < 0.01.

The regression analysis identifies the statistically significant relationships between the above two variables and the standardized mortality rates with similar coefficients (Table 9). The value of *R*^2^ reached 0.637, which could explain the high intensity. However, the proportion to the population aged 15 years and over exhibited no statistical significance among males. Overall, these two factors were significantly associated with the total standardised mortality. We constructed the regression equation as Y = 0.061x_1_−0.065x_2_, where x_1_ denotes the percentage of the uneducated population to the total population aged 15 years and over and x_2_ denotes the percentage of the population with a high school education to the total population aged 6 years and over.

**Table 9:** Regression of educational attainment and mortality rate

Standardized mortality rate	*R*^2^	Percentage of uneducated population to the total population aged 15 y and above (%)			Percentage of the population with a high school education to the total population aged 6 y and above (%)		
**Male**	**Female**	**Total**	**Male**	**Female**	**Total**
**Male**	0.696	0.062			−0.095†		
**Female**	0.533		0.062†			−0.035*	
**Total**	0.637			0.061*			−0.065†

*P < 0.05.

†*P* < 0.01.

### Chinese regional population quality clustering on mortality rate

**Figure 2:**
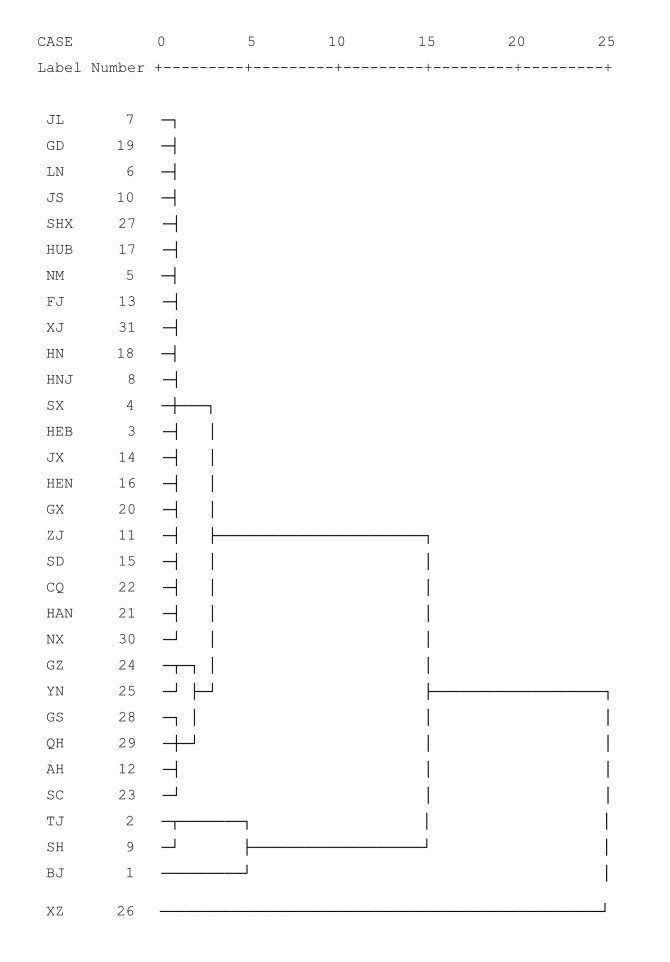
Clustering of Chinese regional population quality based on mortality rate. Class I: Beijing (BJ), Tianjin (TJ), and Shanghai (SH). Class II: Hebei (HEB), Shanxi(SX), Inner Mongolia(NM), Liaoning(LN), Jilin(JL), Heilongjiang(HLJ),Jiangsu (JS), Zhejiang (ZJ), Fujian (FJ), Jiangxi (JX), Shandong (SD), Henan (HEN), Hubei (HEB), Hunan (HN), Guangdong (GD), Guangxi (GX), Hainan (HAN), Chongqing (CQ), Shaanxi (SHX), Gansu (GS), and Xinjiang (XJ). Class III: Anhui (AH), Sichuan (SC), Qinghai (QH), Ningxia (NX), Guizhou (GZ), and Yunnan (YN). Class IV: Tibet (XZ)

After performing double factor clustering based on the results, we determined a classification number of four to be reasonable. The specific classification was as follows. The mortality rate represents population quality: I–IV indicated the decrease in population quality. The highest ratings were obtained in Beijing, Tianjin, and Shanghai, whereas the lowest was obtained in Tibet (**Figure 2**).

## DISCUSSION

### Overall relation between mortality rate and educational attainment

The structure of the educational attainment differs from those in developed countries [6]; educational level was less than that in developed countries. Our findings indicate that, up to 2010, the general trend in the relationship between mortality rate and educational rate China was as follows: the higher the educational level, the lower the crude death rate. This mortality decrease is mainly attributed to the educational levels improvement, which could lead to the improved lifestyles, and work environments. To begin with, higher educational levels typically lead to better jobs and higher incomes, which enable individuals to set aside money to address health concerns [19]. This helps ensure the physical and mental health of educated individuals [20]. Moreover, higher educational levels are believed to influence people’s health awareness and health behavior, thereby enhancing the efficiency of health production (productive efficiency) and efficiency (allocated efficiency) [21].

In this study, we found that the mortality rate of males was higher than that of females with the same education level because of biological, lifestyle, and social environment differences. In general, males have higher basal metabolic rates. In addition, risk factors in traditionally male occupations tend to be higher than those in traditionally female occupations. Compared with women, the lifestyle of men is neither scientific nor healthy. Hence, males are generally less healthy than females, and this, naturally, affects gender-specific mortality rates [22]. However, consistent with previous studies [11], the crude death rate of males decreased more rapidly compared to that of females with increasing educational levels.

We also found that China’s population has an excessively high proportion of older adults. The older adult population has a low educational level, which creates a heavy social burden. The high mortality rate does not truly reflect the illiteracy level of the uneducated population because a high proportion of the older adult population had no education [23], and the mortality rate of the older adult population was relatively high. The original one-child policy was gradually adjusted to balance population development strategies. The two-child policy is a good transition policy implemented at an appropriate time [24].

### Effect of educational level on mortality rate is evident over time

Our study showed that, after controlling for gender, age, and other demographic factors, educational level had a significant effect on health in each given time dimension: a higher educational level was associated with better health and longer life, consistent with existing results [25].

Interestingly, contrasting with other census years, the mortality rate of uneducated males in 2010 increased but declined for uneducated females. This tends to indicate that uneducated males had more difficult lives in 2010 compared with in previous years and that social and education levels are more important for men. Our study indicates that the average ages of both the no education and the primary school populations in 2010 were high, inconsistent with the previous studies [16]. However, the trend is the same: the higher the education level, the lower the mortality rate.

With regard to the mortality gap between 2000 and 1990, between 1990 and 2010, the former was smaller, exhibiting the law of diminishing marginal benefit. The reduction in the standardized mortality rate in populations with primary school and junior high school education was higher than the reductions for other education levels. This suggests the importance of investing more resources to develop the coverage rate for nine-year compulsory education/the graduation to reduce the mortality. In addition, our study does not suggest that city people mortality in the illiteracy is higher than in rural areas and the illiteracy of the population in the city life is more difficult, inconsistent with the finding from Hu Ping's study [11].

### Mortality rate of urban population at different educational levels is lower than rural population

Our study finds that mortality rate of urban population at different educational levels is lower than that of rural population. On the basis of the difference in mortality rates of populations between lower and higher education levels, investments in primary schools and high schools should be increased to achieve positive outcomes. By contrast, no efficiency would be gained from investing in a bachelor’s degree in a city, in a bachelor’s degree or a postgraduate degree in a town, and in junior high school in a village.

### Effects of other educational attainment indicators on mortality rate

This study showed that the percentage of the uneducated population to the total population aged 15 years and over (%) was positively correlated with the mortality rate, whereas the percentage of the population with a high school education to the total population aged 6 years and over (%) was negatively correlated with the mortality rate. The value of R^2^ was 0.637 for the two-factor regression and we were able to explain the main variance in mortality rate for different levels. The government should place considerable importance on the contribution of education to health indicators, as represented by mortality rate.

### Population quality varies by region

Inequality in socioeconomic status (SES) is common in China, and the government should therefore prioritize reducing inequities in education [26]. In this study, we divided population quality into four categories. The government can adjust training policies and reduce regional differences to promote social equity by referring to population quality. Different regions will also be aware of their situations and implement measures to improve the quality of their populations including improving educational attainment and reducing mortality rates.

### Limitations

We believe the under-reported mortality, if any, is minimized in the strictly conducted national population Census. Although the best approach is direct standardization, the absence of key data forced this study to mostly apply an indirect standardization method to its subjects. Ecological fallacy may exist, though this study assesses the relationship between education and mortality from a holistic perspective. Other confounding factors may have not been ruled out, and thus, relevant regression results should only be used as a reference, and not be regarded as being conclusive. However, the analysis produced a high decision coefficient, suggesting the validity of our findings.

Education is an endogenous variable, with a reverse causality to health. However, the causal relationship between health and education is difficult to determine. Although rigorous analyses have been conducted to gradually confirm this causal relationship [8], further research is needed to confirm the independent(causes) and dependent(outcome) factors. In most studies in China, influence is determined based on the compulsory education law or sudden disasters (eg, widespread famine). At present, the two-child policy in China could also serve an exogenous variable for considering the causal relationship between health and education. In addition, given the strong associations between educational level and mortality rates, the inclusion of more detailed questions on education in China health surveys and population censuses remains important.

## CONCLUSIONS

We found that educational level was negatively correlated with the mortality rate. Together with previous research findings, this study indicates that improving total population education attainment remains an important challenge that requires immediate action. Reducing education inequities is particularly crucial for the government.
